# The Profile of the Brazilian Cardiologist - A Sample of Members of the Brazilian Society of Cardiology

**DOI:** 10.5935/abc.20190089

**Published:** 2019-07

**Authors:** Lucas Simonetto Faganello, Mauricio Pimentel, Carisi Anne Polanczyk, Tiago Zimerman, Marcus Vinicius Bolivar Malachias, Oscar Pereira Dutra, Leandro Ioschpe Zimerman

**Affiliations:** 1 Hospital de Clínicas de Porto Alegre, Porto Alegre, RS - Brazil; 2 Faculdade de Ciências Médicas de Minas Gerais, Belo Horizonte, MG - Brazil; 3 Instituto de Cardiologia - Fundação Universitária de Cardiologia do Rio Grande do Sul, Porto Alegre, RS - Brazil

**Keywords:** Cardiologists, Survey and Questionnaires, Income, Gender, Demographic Data, Quality of Life

## Abstract

**Background:**

Data from the international literature have shown changes in the profile of cardiologists and in their medical practices. However, there is no data on this in Brazilian cardiologists.

**Objective:**

To evaluate professional and personal characteristics of a sample of Brazilian cardiologists.

**Methods:**

This was a cross-sectional study; a questionnaire was sent by e-mail to cardiologists, active members of the Brazilian Society of Cardiology in 2017. The results were analyzed, and the level of significance set at p < 0.05.

**Results:**

The questionnaire was sent to 13,462 cardiologists, with 2,101 (15.6%) respondents, mostly men (71.8% versus 28.2%). Age distribution and marital status were significantly different between the sexes (p < 0.001). The number of cardiologists without children was higher among women (40.5% versus 16.1%; p < 0.001). The most common place of work was the public hospital (46.5%), followed by private hospital (28.5%) and private office (21.1%). The office was the main place of work for 23.9% of men and 14% of women (p < 0.001), with predominance of individuals older than 50 years (31.7% versus 10.1%, respectively; p < 0.001). Most cardiologists (64.2%) worked more than 40 hours a week (69% of them men and 51.9% of the women; p < 0.001). Eighty-eight percent of the sample earned more than BRL 11,000 (US$ 3,473.43), and 66.5% of the men earned more than BRL 20,000 (US$ 6,315.32) per month, versus 31.2% of the women (p < 0.001). A high level of work-related stress was reported by 11.3% of respondents.

**Conclusion:**

Most cardiologists were men, who showed higher workload and higher income; 11.3% of the cardiologists perceived stress as a great deal.

## Introduction

Medical activity has been associated with high levels of stress and dissatisfaction compared with other occupations.^1^ Previous studies have shown that there is a complex relationship between the level of work satisfaction, work-life balance, burnout level, demographic factors and work conditions.^2^

The progress of medicine and specialties, especially in the last decades, has promoted rapid chances in personal and professional lives of cardiologists.^3-5^ In addition, international data have shown the aging of working classes, with changes in the profile and characteristics of professional activities, but still a predominance of men and a striking difference of salaries between the sexes.^6^ However, data on the profile of Brazilian cardiologists and their perceptions of the profession are limited.

Therefore, the present study describes the profile of the Brazilian cardiologist from data obtained in the study conducted by the Brazilian Society of Cardiology (BSC), in terms of demographic, social, and professional aspects and quality of life.

## Methods

The BSC developed a questionnaire composed of three main domains: demographic data, professional activity and quality of life. This questionnaire was sent by e-mail to 13,462 cardiologists, active members of the BSC in 2017. A total of 2,101 participants answered the questionnaire and composed the study group, 1,509 men (71.8%) and 592 women (28.2%).

### Statistical analysis

The variables were expressed as absolute frequency, percentage and 95% confidence interval. Qualitative variables were compared using the chi-square test followed by analysis of adjusted standardized residuals. Analysis of the answers was performed using the SPSS Statistics for Windows version 25.0 (IBM, Chicago). The level of significance was set at 5% (p < 0.05).

## Results

### Demographic data

Of the 13,462 active members of the BSC, 9,555 (70.9%) were men, 9,752 (71.1%) were aged between 30 and 59 years, and 1,323 (9.8%) aged 70 years or older. Most members lived in the southeast (55.5%) or in the south (15.1%) of Brazil as follows - 3,420 (25.4%) in São Paulo, 2,158 (16%) in Rio de Janeiro, 1,572 (11.6%) in Minas Gerais, 853 (6.3%) in Rio Grande do Sul and 774 (5,7%) in Paraná State.

**Figure 1:**
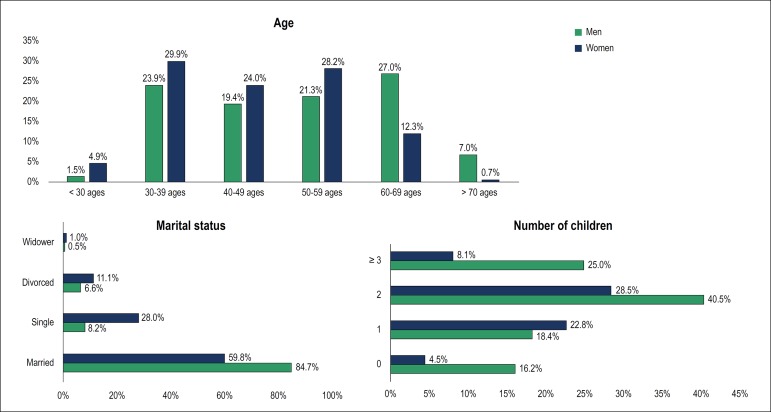
Demographic characteristics.

The questionnaire was answered by 2,101 cardiologists (response rate 15.6%), 1,509 men (71.8%) and 592 women (28.2%). Regarding the age of participants, 1,077 (51.3%) were older than 50 years. Figure 1 shows the distribution of age by sex, with a difference between men and women (p < 0.0001). Thirty-four percent of men and 13% of women were aged 60 years or older. Most respondents were married (77.7¨%), 84.7% of the men and 59.8% of the women (p < 0.001). With respect to the number of children, 23% did not have children, and 57.3% had two or more children. The number of cardiologists who did not have children was higher among women (40.5 *versus* 16.1%; p < 0.001). The difference remained significant after adjustment for age, but not after adjustment for marital status.

### Characteristics of professional activity

Most respondents (70.5%) had the title of specialists in cardiology from the BSC/Brazilian Medical Association, and 29.5% were applicants for the title. Most participants (n = 1,336; 65.4%) worked in only one field, especially clinical cardiology (50.5%), with no difference between men and women; 2.8% of the interviewees did not answer this question.

**Figure 2:**
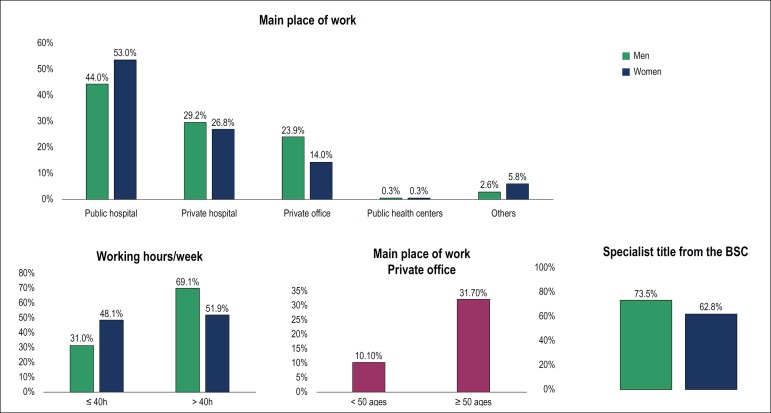
Professional characteristics. BSC: Brazilian Society of Cardiology

Regarding the number of working hours per week, 1,350 (64.2%) participants (69% of the men and 51.9% of the women, p < 0.001) worked more than 40 hours; 363 (17.3%) worked in only one place, and 1,036 (49.3%) worked in three or more places. The most common place of work was the public hospital (46.5%), followed by private hospital (28.5%) and private office (21.15) (Figure 2). The public hospital was the main place of work for 53% of women 44% of men (p<0.001). On the other hand, private office was the main work place for 23.9% of men and 14% of women, three times more frequent among cardiologists older than 50 years (31.7 versus 10.1%). Only 0.3% of participants reported to work in public health centers. No difference was found in the number of work places between the sexes.

**Figure 3:**
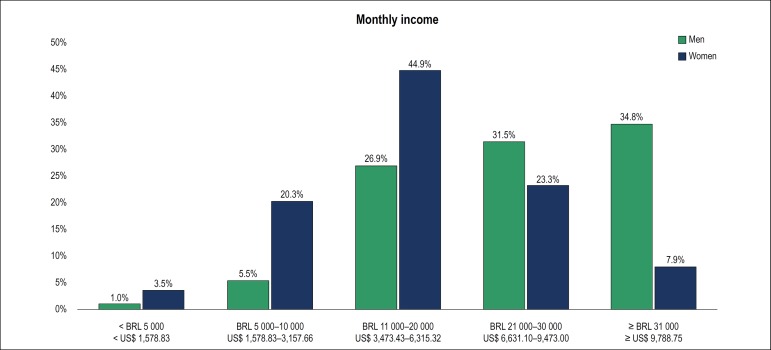
Salary difference between men and women.

Monthly income was higher than 11,000 Brazilian reals (BRL) (US$ 3,473.43). The distribution of income by sex is depicted in Figure 3; 66.5% of men and 31.2% of women (p < 0.001) reported to gain more than 20,000 BRL (adjusted for number of working hours and age range).

**Figure 4:**
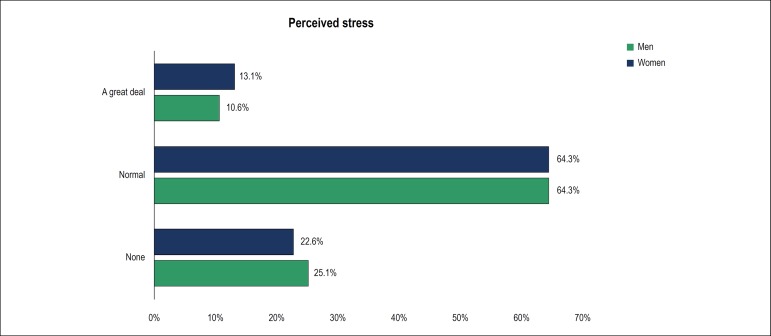
Level of stress.

Regarding the level of stress (Figure 4), 64.2% reported an adequate level of stress; 11.3% perceived stress as a great deal, and 24.3% reported no stress. The causes of stress at work were poor working conditions (36.7%), excessive working hours (23.5%), low pay (15.7%), work pressure (10.9%), and others (13%).

With regards to legal issues, 13.9% of participants reported having been sued for medical malpractices, and 0.3% reported to have been condemned. The question was not answered by 10 participants (0.4%).

Considering the age participants wished to retire, 10.5% reported they wished to retire before the age of 60 years, 46.1% between 61 and 70 years, 34.1% between 71 and 80 years, 4.7% older than 80 years, and 14.6% reported they do not want to retire from their work as a cardiologist. Regarding the retirement financial planning, 58.4% reported to contribute to a private retirement plan.

The questionnaire also included questions about participants' perception on the influence of technology, spirituality, the role of the media on their work. The use of technology on a daily basis was reported by 84.7% of the respondents, with no difference between sexes and age ranges. The frequency of technology use for patient-physician communication was daily for 40.6% and weekly for 21.7% of participants. Regarding the role of spirituality in the treatment of the patients, it was considered "very useful" by 54.7%, "useful" by 30.1%, "not very useful" by 6% of the respondents; 9.1% did not have a strong opinion about the issue, and 0.5% did not answer the question. In terms of the frequency the physicians talked about the subject "spirituality" with their patients, 54.1% answered "eventually", 24.5% "often", 14.2% "never" and 0.5% did not answer the question. As for how medical work was presented by the media, 73.7% considered that the opinion expressed by the media was partial and unfavorable to the doctors.

### Quality of life

**Figure 5:**
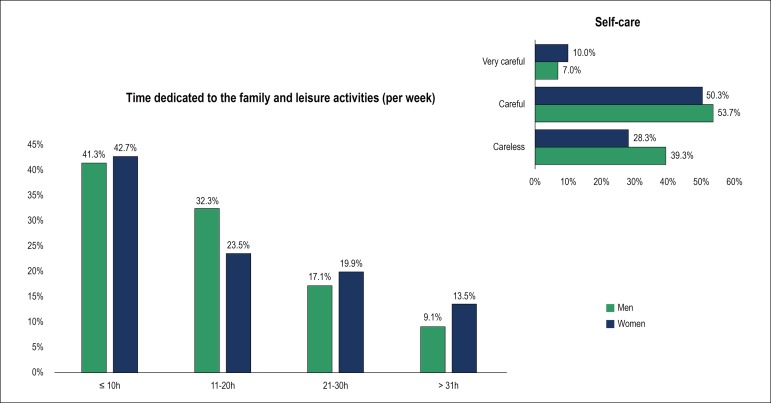
Quality of life.

Most cardiologists reported to sleep between 6 and 7 hours per night (79.5%), with no differences between men and women. Considering the time dedicated to family and leisure activities (Figure 5), 41.8% answered less than 10 hours per week; 29.8% between 11 and 20 hours per week, 17.9% between 21 and 30 hours per week, and 10.4% 31 hours or more per week. The proportion of women who spent more than 20 hours with family-related and leisure activities was greater than that of men (33.4% *versus* 26.2%; p < 0.001).

Smoking habit was reported by only 2.8% of cardiologists (3.3% of men and 1.5% of women, p = 0.03). Regarding alcohol consumption, 34.3% reported the frequency of ≥ 2 times (40.1% of the men and 19.5% of the women, p,0.001), 30.1% sometimes a month, 23.4% rarely, and 12% never. Regarding health-related self-care, 52.6% considered themselves to be careful, 7.9% very careful, and 3.4% not careful, with no difference between men and women. With respect to the health of participants, 56.1% reported to have a disease (42.5% of the men and 63.3% of the women; p < 0.001). Sixty percent reported continuous use of medication (57.3% of the men and 59.1% of the women, p = 0.46).

## Discussion

This is the third time the BSC developed studies aimed at evaluating the profile of its member associates. However, this is the first time the Society promoted a more comprehensive study and published the results.

Despite the increase of women among medical professionals,^7^ there is a remarkable predominance of men among Brazilian cardiologists, similar to what is observed in other countries.^6,8,9^ Most respondents in our study was men (71.8% versus 28.2%), which is in agreement with the 2018 medical demography in Brazil,^7^ showing a distribution of 69.7% of and 30.3% of women, and mean age of 48.9 ± 12.1 years among Brazilian cardiologists. This difference is greater among cardiologists older than 50 years and less pronounced in younger subjects. Differences between sexes were observed for marital status, number of children, income and workload.

The proportion of married men who answered the questionnaire was significantly higher than that of women. The proportion of cardiologists who did not have children was higher among women, even after adjustment for age. Such difference is in accordance with the results of a study conducted with North-American cardiologists.^6^ Therefore, it is possible to infer that the prolonged time from graduation and specialization may be associated with these findings.

Our study showed that, in higher income ranges, the proportion of men was higher than of women, adjusted for work load and number of workplaces. This difference in income between sexes has been reported in several other professional sectors^5^ and has been registered in the last decades among North-American cardiologists. Most professionals reported to work more than 40 hours a week, with higher proportion of men in the private sector. The proportion of women was higher in the public and academic settings, in which a lower income was identified as compared with the private setting.

Almost half of cardiologists work in three or more places. Interestingly, the private office was reported to be the main place of work by only 21.1% of the respondents, and this rate was even lower when considering individuals younger than 50 years only. Thus, cardiology practice in private sectors has been less commonly seen probably due to elevations in the number of doctor visits covered by private health plans. These plans pay a relatively low price per service to the physicians, who feel discouraged to set up and maintain their own clinics. This change in the professional scenario may also be related to the fact that younger cardiologists are concerned not only about seeking more immediate revenue but also about investing in complementary pension.

Despite the high work load, only 11.3% of cardiologists perceived stress as a great deal. A study published by Mescape^10^ concluded that 46% of North-American cardiologists had symptoms of burnout, similar to the frequency reported in other specialties like pneumology and nephrology. Although the questionnaire used in the study did not include specific criteria for the diagnosis of burnout, data have suggested that there are considerable differences between Brazilians and North-Americans regarding the perception of stress at work, which can be related to sociocultural differences. Also, there is a relatively lower occurrence of medical lawsuits among Brazilian cardiologists. While 0.3% of our sample reported having been condemned, a study published in the American Heart Journal in 2014^11^ reported a rate of malpractice lawsuits of 8.6% in North America and a 1% condemnation rate.

The practice of physical exercise has well-established cardiovascular benefits and should be encouraged. In our study, however, 31% of cardiologists did not practice exercise. Only 11% of North-American cardiologists reported to be physically inactive.^10^ This difference may be due to the higher workload faced by physicians in Brazil. Although the percentage of smokers was relatively low, a high percentage of cardiologists reported they were careless about their health (39.4%). Regarding alcohol consumption, 34.3% of respondents reported a frequency of two times a week or more; 49% of North American cardiologists consumed up to one drink per week. These data indicate that institutional campaigns aimed at promoting the adoption of healthy habits and self-care among Brazilian cardiologists should be considered.

The use of technology has facilitated the search for knowledge and contributed in different aspects of daily life; also, it has allowed patients to communicate more directly with their doctors. In our study, most cardiologists reported to use any kind of technology on a daily basis in their work. A significant percentage (40.6%) of cardiologists is contacted daily by patients through technology device, which adds to the debate about the availability of physicians to respond to patients' emails or on social media.

According to the Brazilian Medical Council's resolution, published in 2002,^12^ despite numerous positive consequences of telemedicine, it is related to serious ethical and legal issues. Considering the exponential use of social media by physicians, including for patient-doctor communication, the Medical Council, in 2017,^13^ recognized the importance of the social media and stablished rules of conduct in this context.

There has been increasing interest in issues of spirituality in the medical practice. In the BSC, a group of study on spirituality and cardiovascular medicine was created. In the present study, although 54.7% of participants considered that spirituality was very useful in the treatment of the patients, 14.2% has never talked about this subject with their patients and most (54.1%) of them did it only eventually. This difference may be related to the scarcity of validated qualification in the area.

The study has some limitations, particularly regarding data collection. The rate of response to the questionnaire was relatively low (15.6%), and on this basis, we cannot affirm that the results would be the same in a larger sample. However, similar studies have shown comparable results (15-25%). In the most recent study conducted by the American College of Cardiology, which has continuously developed studies on the theme in the last three decades, the response rate was 21%. In the study conducted by the Medscape, the rate of response was 4%.

## Conclusion

In this original study on the profile of Brazilian cardiologists, associates of the BSC, significant differences between sexes were detected. While men had a greater work load, the participation of women in higher income ranges was lower, even after adjustment for work load. Most cardiologists worked in more than one place, mostly in the public sector. Younger cardiologists had a lower tendency to work in private offices. The perception of the level of work-related stress was considered satisfactory. Considering the growing concern over personal and professional quality of life, as well as over work performance, future research is warranted to further explore the theme and to allow the planning of actions by medical entities and societies, so that the specialty of cardiology can continue to attract the interest of medical professionals.
